# Distinguishing Coronavirus Disease 2019 Patients From General Surgery Emergency Patients With the CIAAD Scale: Development and Validation of a Prediction Model Based on 822 Cases in China

**DOI:** 10.3389/fmed.2021.601941

**Published:** 2021-04-30

**Authors:** Bangbo Zhao, Yingxin Wei, Wenwu Sun, Cheng Qin, Xingtong Zhou, Zihao Wang, Tianhao Li, Hongtao Cao, Yujun Wang, Weibin Wang

**Affiliations:** ^1^Department of General Surgery, State Key Laboratory of Complex Severe and Rare Diseases, Peking Union Medical College Hospital, Chinese Academy of Medical Sciences, Peking Union Medical College, Beijing, China; ^2^Department of Critical Care Medicine, The Central Hospital of Wuhan, Tongji Medical College, Huazhong University of Sciences and Technology, Wuhan, China; ^3^Department of Breast Surgery, State Key Laboratory of Complex Severe and Rare Diseases, Peking Union Medical College Hospital, Chinese Academy of Medical Sciences, Peking Union Medical College, Beijing, China

**Keywords:** COVID-19, infectious acute abdomen, prediction model, nomogram, prediction scale

## Abstract

**Background:** During the epidemic, surgeons cannot identify infectious acute abdomen patients with suspected coronavirus disease 2019 (COVID-19) immediately using the current widely applied methods, such as double nucleic acid detection. We aimed to develop and validate a prediction model, presented as a nomogram and scale, to identify infectious acute abdomen patients with suspected COVID-19 more effectively and efficiently.

**Methods:** A total of 584 COVID-19 patients and 238 infectious acute abdomen patients were enrolled. The least absolute shrinkage and selection operator (LASSO) regression and multivariable logistic regression analyses were conducted to develop the prediction model. The performance of the nomogram was evaluated through calibration curves, Receiver Operating Characteristic (ROC) curves, decision curve analysis (DCA), and clinical impact curves in the training and validation cohorts. A simplified screening scale and a management algorithm were generated based on the nomogram.

**Results:** Five potential COVID-19 prediction variables, fever, chest CT, WBC, CRP, and PCT, were selected, all independent predictors of multivariable logistic regression analysis, and the nomogram, named the COVID-19 Infectious Acute Abdomen Distinguishment (CIAAD) nomogram, was generated. The CIAAD nomogram showed good discrimination and calibration, and it was validated in the validation cohort. Decision curve analysis revealed that the CIAAD nomogram was clinically useful. The nomogram was further simplified as the CIAAD scale.

**Conclusion:** We established an easy and effective screening model and scale for surgeons in the emergency department to use to distinguish COVID-19 patients. The algorithm based on the CIAAD scale will help surgeons more efficiently manage infectious acute abdomen patients suspected of having COVID-19.

## Background

Since the outbreak of coronavirus disease 2019 (COVID-19), which was characterized as a pandemic by the World Health Organization on March 11, 2020, the virus has rapidly spread globally (1, 2). Millions of people have been infected, resulting in tens of thousands of deaths (3). The ongoing pandemic is not only an enormous threat to public physical health but also an acid test for the medical systems in both developed counties and developing countries (4). In addition to prevention, the quick and accurate recognition of COVID-19 is currently one of the most important tasks.

The medical management of other diseases has been critically disturbed, especially in diseases characterized by fever, which is a typical symptom of COVID-19 (5). There are numerous high-risk people who are coming into close contact with confirmed COVID-19 patients. Disrupting transmission is the most effective way to control the epidemic of COVID-19. Under the current situation, an infectious acute abdomen is still one of the most common surgical emergencies. Patients with acute abdomen infection often display fever and similar changes in routine blood and other biochemistry tests as those observed in patients with COVID-19, which would cover up the signs of COVID-19 (6). Currently, the diagnosis of COVID-19 mainly depends on severe acute respiratory syndrome coronavirus 2 (SARS-CoV-2) nucleic acid detection (7). However, that standard procedure is to double-check, which is time-consuming and still has the risk of false-negative results (8), and this hinders efforts to perform emergency operations in a timely manner and to prevent cross-infection in the hospital. Therefore, there is a pressing need for an easier and more feasible method to help surgeons preliminarily distinguish COVID-19 patients from other infectious acute abdomen patients who have symptoms mimicking those of COVID-19 and take proper precautions in emergency operations.

Using the clinical data of 822 patients, 584 with COVID-19 and 238 with infectious acute abdomen cases, we compared the demographic, clinical, imaging, and laboratory characteristics to identify the significant predictors of COVID-19. Furthermore, a prediction model to distinguish between the two diseases was generated based on machine learning and is presented in the form of a nomogram, which had good discrimination performance in both the training and validation cohorts. Ultimately, we offer a practical screening scale, named the CIAAD scale, and an algorithm, with accompanying precautionary advice for surgeons treating infectious acute abdomen patients.

## Methods

### Patients

Ethics approval was obtained from the Ethics Committees of A Hospital (Note. a hospital in Wuhan, name omitted for review) and the B Hospital (Note. a hospital in Beijing, name omitted for review) for this retrospective study. We included 584 COVID-19 patients seen at the A Hospital between January 2, 2020, and February 15, 2020, in our study. The diagnostic criteria for COVID-19 were positive RT-PCR results for SARS-CoV-2 or viral gene sequencing results that were highly homologous with SARS-CoV-2 using respiratory or blood samples (7). Since the routine medical treatment of other diseases in Wuhan was severely disturbed by the epidemic, the clinical data of 283 infectious acute abdomen patients undergoing emergency operations at B Hospital between February 28, 2019, and April 3, 2020, were collected. The definition of infectious acute abdomen cases was an acute abdomen with a primary infectious cause, such as acute appendicitis, or with secondary infectious peritonitis, such as perforation, obstruction, and bleeding. The inclusion criteria were as follows: (1) fever; (2) abnormal routine blood results or other infection indicators; or (3) signs of pneumonia. The patients with infectious acute abdomen admitted after January 20, 2020, were all tested for SARS-CoV-2 and none of them were positive. Those admitted before January 20 were not tested for SARS-CoV-2 and assumed as negative as the epidemic had not broken out yet.

### Data Collection and Definitions

Demographic, clinical, laboratory, treatment, and outcome data from the COVID-19 and infectious acute abdomen patients were extracted from the electronic medical records system of the A Hospital and B Hospital, respectively. The ranges of normal values and definitions for different variables were the same.

Fever was defined as an axillary temperature of at least 37.3°C. The chest CT scores were graded retrospectively by two radiologists in sequence. Each lung was divided into the upper, middle, and lower lobes, and the scoring criteria were as follows: <1/3 of the lung infected, 0 points; 1/3–2/3 of the lung infected, 1 point; >2/3 of the lung infected, 2 points. The classification of COVID-19 severity was based on the Chinese management guidelines for COVID-19 (version 7.0) published by the National Health Commission of China (7).

### Potential Predictor Selection

The primary cohort of all 822 patients was randomly divided into a training cohort and a validation cohort at a ratio of 2:1. The least absolute shrinkage and selection operator (LASSO) method, one of the most effective methods of regularized regression with substantial advantages when managing multicollinear data, was used to select the most useful predictive variables for COVID-19 in the training cohort (9).

### Development and Validation of a Prediction Model

We conducted multivariate logistic regression with the potential predictors to further verify their predictive efficacy and then built a nomogram to distinguish COVID-19 patients from infectious acute abdomen patients on the basis of the results of the multivariable logistic analysis in the training cohort. We named the nomogram the COVID-19 and Infectious Acute Abdomen Distinguishment (CIAAD) nomogram. Calibration curves were plotted to assess the calibration of the CIAAD nomogram, and the C-index was measured to quantify its discrimination performance. Then, the CIAAD nomogram developed using data from the patients in the training cohort was applied to the patients in the validation cohort, and the calibration curve and C-index were derived on the basis of the regression analysis. The ability of the CIAAD nomogram to distinguish between the two groups of patients in both the training and validation cohorts was also assessed by calculating the area under the receiver operating characteristic curve (AUC).

### Clinical Usefulness Assessment

Decision curve analysis was performed and clinical impact curves generated to evaluate the clinical practicability of the CIAAD nomogram by quantifying the net benefits at different threshold probabilities in both the training and validation datasets.

### Development of Screening Scale

The score for each item in the CIAAD nomogram was divided by 25 and then rounded to obtain a simplified score. The simplified scores were verified to have the same efficacy as the original nomogram. We subdivided the risk of COVID-19 into low (<0.3), moderate (0.3–0.7), and high (>0.7) risk. The CIAAD Scale was generated on the basis of the simplified scoring criteria and risk classification.

### Statistical Analysis

Categorical variables are expressed as numbers and percentages. Continuous variables are expressed as medians with interquartile ranges. The chi-square test and Mann-Whitney U-test were used to evaluate categorical and continuous data, respectively. Statistical analysis was conducted with R software (version 3.6.1; http://www.Rproject.org) and the SPSS statistical software package (version 25.0). *P* < 0.05 was considered statistically significant.

## Results

### Demographic and Clinical Characteristics

**Figure 1:**
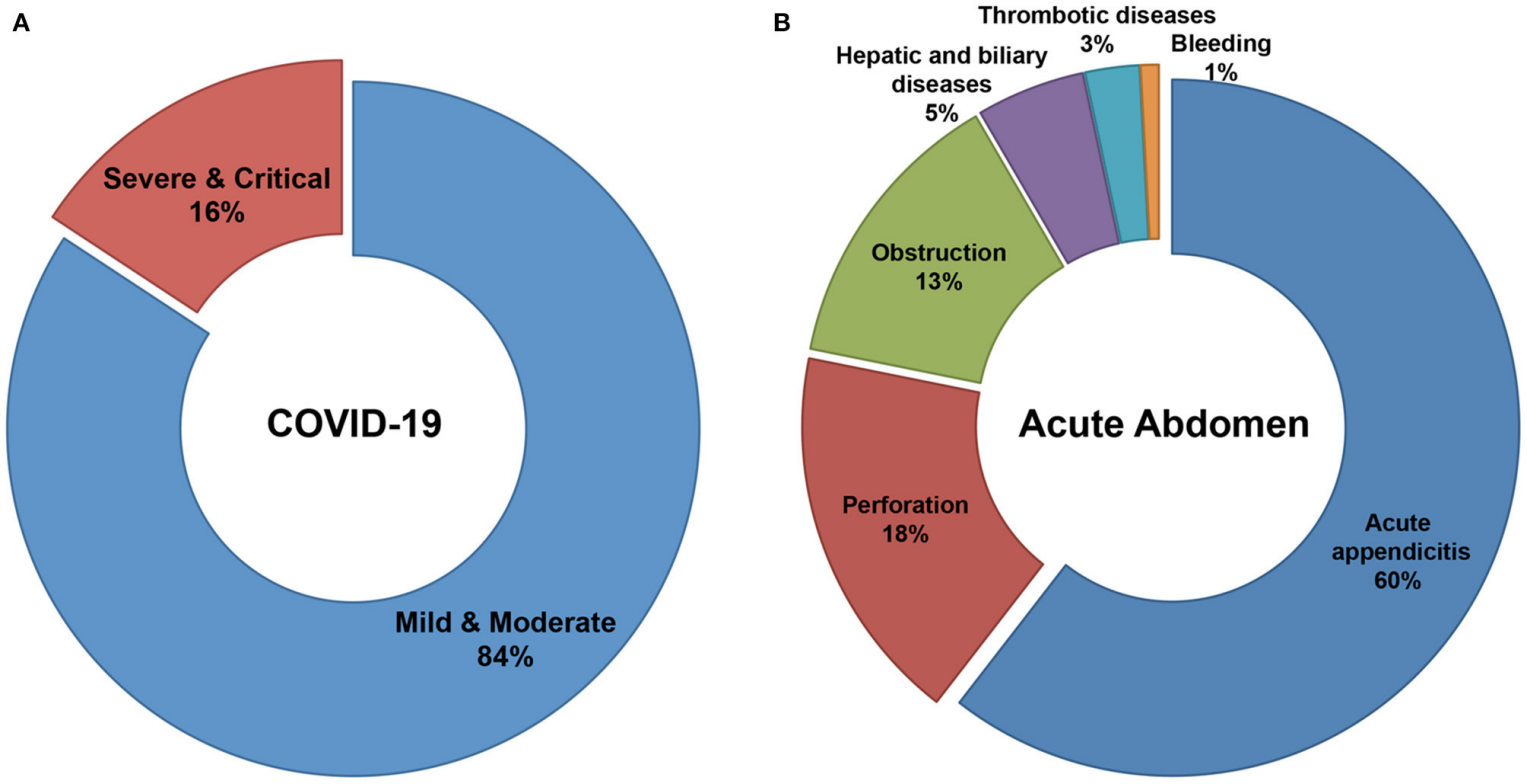
Distribution of severity in COVID-19 patients and disease spectrum in infectious acute abdomen patients. **(A)**. 16% of enrolled COVID-19 patients were severe or critical and the left 84% were mild or moderate. **(B)**. The disease spectrum of enrolled infectious acute abdomen patients showed the top three causes for emergency operations were acute appendicitis (60%), gastrointestinal perforation (18%), and bowel obstruction (13%).

A total of 822 patients, 584 COVID-19 patients without infectious acute abdomen and 238 infectious acute abdomen patients without COVID-19, were included in this study (Table 1). Nearly 16% of the COVID-19 patients had severe or critical COVID-19 (Figure 1A). The infectious acute abdomen patients primarily had acute appendicitis (60.5%), perforation (17.6%), and obstruction (13.4%) (Figure 1B). Compared with the COVID-19 patients, the infectious acute abdomen patients were younger (*p* = 0.001) and had fewer chronic diseases, such as diabetes (*p* = 0.005) and cardiovascular and cerebrovascular diseases (*p* = 0.011). Fever, as reported before, was the most common symptom in the COVID-19 patients (80.1%), and nearly one-third of the infectious acute abdomen patients got abnormal body temperature as well. COVID-19 was associated with a larger infected proportion of the lung (*p* < 0.001). Nevertheless, the abdominal infection causing acute abdomen resulted in more abnormal laboratory test results, such as CRP, PCT, WBC, neutrophils, and fibrinogen, in infectious acute abdomen patients than in COVID-19 patients (*p* < 0.001).

**Table 1:** Demographic, clinical, imaging, and laboratory characteristics of patients on admition or first to emergency.

**Clinical variables**	**All patients (*n* = 822)**	**COVID-19 (*n* = 584)**	**Acute abdomen (*n* = 238)**	***P*-value**
Age (years), No. (%)	53.0(36.0–66.0)	55.5(38.0–67.0)	46.5(33.8–64.0)	0.001
Gender				0.012
Male, No. (%)	386(47.0)	258(44.2)	128(53.8)	-
Female, No. (%)	436(53.0)	326(55.8)	110(46.2)	-
**Chronic diseases**				
Chronic obstructive pulmonary disease, No. (%)	47(5.7)	39(6.7)	8(3.4)	0.063
Hypertension, No. (%)	251(30.5)	190(32.5)	61(25.6)	0.051
Diabetes mellitus, No. (%)	104(12.7)	86(14.7)	18(7.6)	0.005
Cardiovascular and cerebrovascular diseases, No. (%)	97(11.8)	78(13.4)	19(8.0)	0.011
Renal failure, No. (%)	39(4.7)	33(5.7)	6(2.5)	0.056
**Symptoms**				
Fever, No. (%)	545(66.3)	468(80.1)	77(32.4)	<0.001
Shortness of breath, No. (%)	232(28.2)	228(39.4)	4(1.7)	<0.001
Fatigue, No. (%)	254(30.9)	213(36.5)	41(17.2)	<0.001
Muscle pain, No. (%)	154(18.7)	147(25.2)	7(2.9)	<0.001
Diarrhea, No. (%)	66(8.0)	52(8.9)	14(5.9)	0.148
Chest CT				<0.001
0, No. (%)	492(63.4)	313(55.1)	179(86.1)	-
1, No. (%)	148(19.1)	120(21.1)	28(13.5)	-
2, No. (%)	136(17.5)	135(23.8)	1(0.5)	-
**Infection-related biomarkers**				
CRP level, median (IQR), mg/L, NR^*^ 0–8	19.0 (4.2–48.0)	16.3 (3.6–44.3)	32.0(12.0–120.8)	<0.001
PCT level, median (IQR), ng/mL, NR 0–0.5	0.06(0.04–0.12)	0.05(0.04–0.09)	0.78(0.11–10.25)	<0.001
**Blood routine**				
Leucocytes, median (IQR), × 10^9^, NR 3.50–9.50	5.7(4.1–9.9)	4.9(3.8–6.5)	12.1(9.2–15.5)	<0.001
Neutrophils, median (IQR), × 10^9^, NR 2.00–7.50	4.0(2.6–7.9)	3.3(2.2–4.8)	10.3(7.0–13.7)	<0.001
Lymphocytes, median (IQR), × 10^9^, NR 0.80–4.00	1.0(0.7–1.4)	1.0(0.7–1.4)	1.1(0.7–1.8)	0.02
Platelets, median (IQR), × 10^9^, NR 100–350	189.0(147.0–243.0)	178.0(134.0–224.8)	227.0(181.0–274.8)	<0.001
Hemoglobin, median (IQR), g/L, NR 120–160 (male), 110–150 (female)	131.0(120.0–143.0)	128.0(119.3–140.0)	137.0(120.8–153.0)	<0.001
**Blood biochemistry**				
Alanine aminotransferase, median (IQR), U/L, NR 9–50 (male), 7–40 (female)	18.0(12.0–30.4)	19.7(13.2–32.7)	15.0(9.0–24.0)	<0.001
Total bilirubin, median (IQR), μmol/L, NR 5.1–22.2	9.7(7.1–14.4)	8.6(6.4–11.9)	14.1(9.9–21.5)	<0.001
Blood urea nitrogen, median (IQR), mmol/L, NR 2.78–7.14	4.3(3.3–5.9)	4.1(3.2–5.5)	5.1(3.8–6.9)	<0.001
Serum creatinine, median (IQR), μmol/L, NR 59–104 (male), 45–84 (female)	67.1(54.3–82.3)	64.8(51.8–78.3)	74.0(64.0–93.5)	<0.001
**Coagulation function**				
Fibrinogen, median (IQR), g/L, NR 1.80–3.50	3.1(2.5–3.7)	3.0(2.5–3.5)	3.5(2.8–4.6)	<0.001
D-dimer, median (IQR), mg/L, NR 0–0.55	0.6(0.3–1.5)	0.5(0.3–1.1)	0.8(0.4–3.2)	<0.001

* *NR: normal range*.

### Potential Predictor Selection

**Figure 2:**
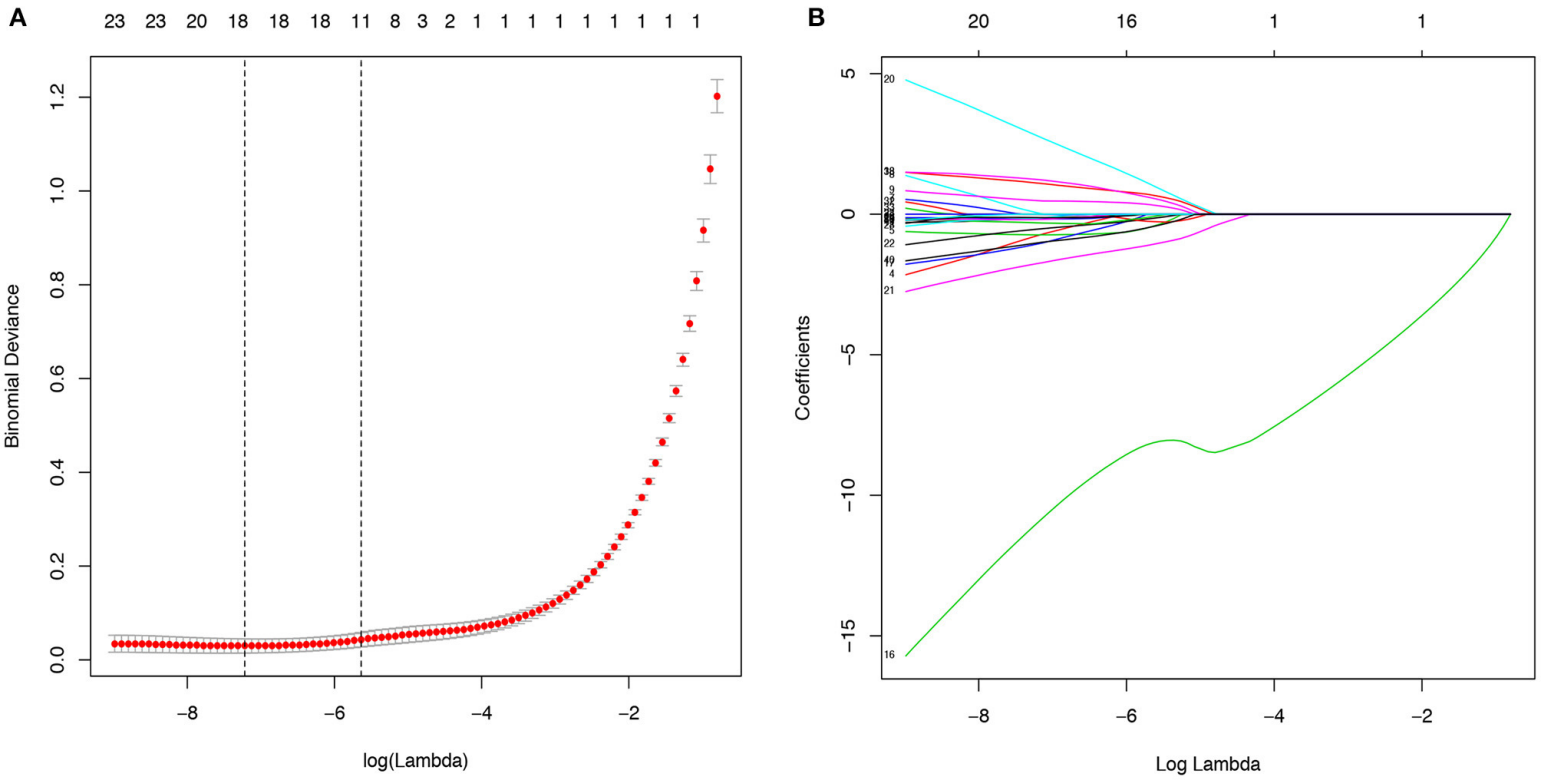
Potential predictors selection using the least absolute shrinkage and selection operator (LASSO) regression model. **(A)**. The binomial deviance curve was plotted versus log(λ). Dotted vertical lines were drawn at the optimal values by using the minimum criteria and the one standard error of the minimum criteria (the 1-SE criteria). **(B)**. LASSO coefficient profiles of the 40 alternative variables. A coefficient profile plot was produced against the log (λ) sequence.

The 38 variables collected were reduced to five potential predictors (abdominal pain, fever, chest CT, CRP, PCT, and WBC) with non-zero coefficients in LASSO regression on the basis of the data from the 547 patients in the training cohort (Figure 2). The AUC of the five variables, namely, fever, chest CT, CRP, PCT, and WBC, was obtained in the training cohort and validation cohort (Supplementary Figures 1, 2). CRP got the highest AUC in both training and validation cohorts of 0.835 and 0.854.

### Development of a Prediction Model

**Figure 3:**
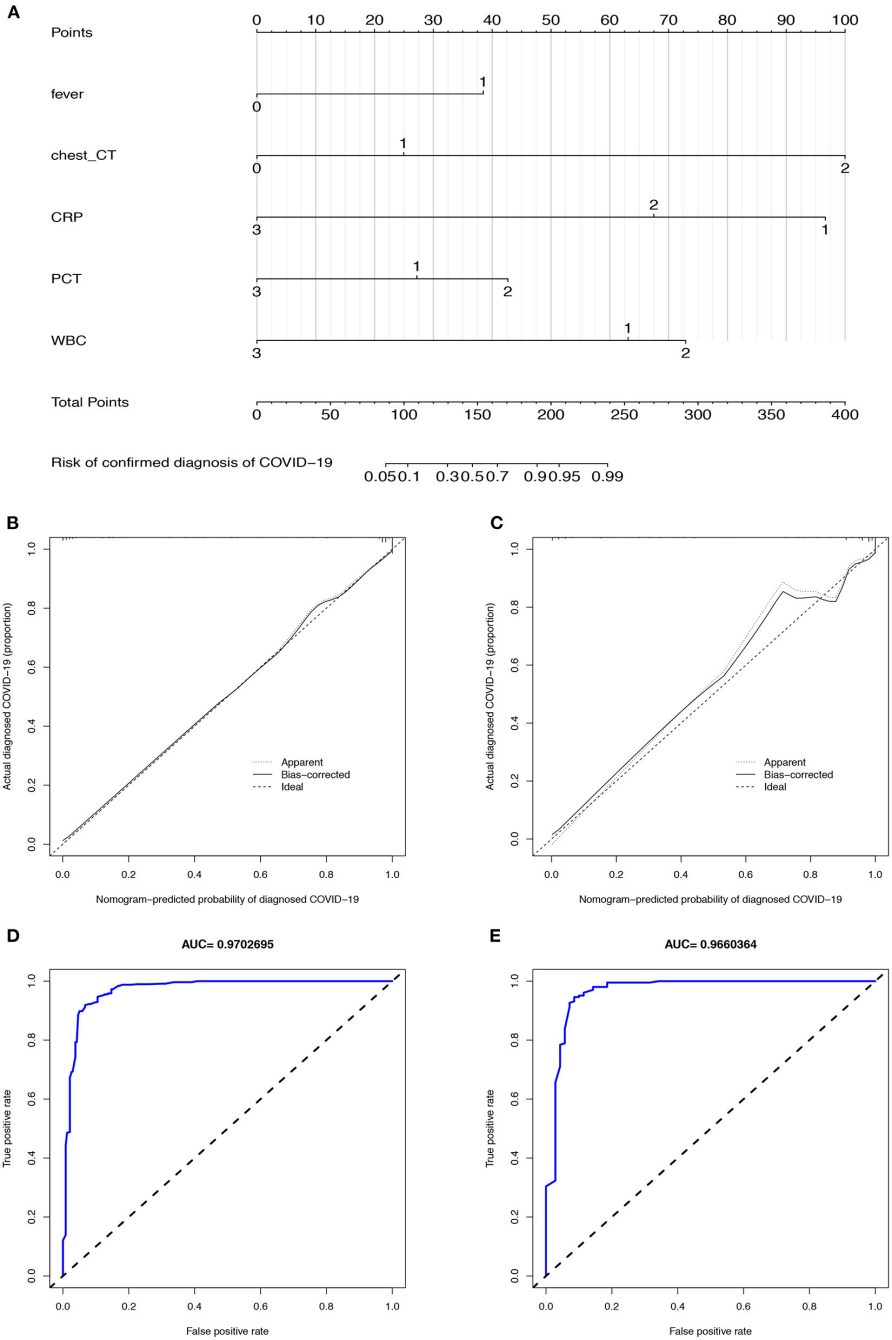
The CIAAD nomogram and its discrimination performance in training and validation cohort. **(A)**. The CIAAD nomogram was developed in the training cohort based on fever, chest CT, CRP, PCT, and WBC. **(B)**. Calibration curve of the CIAAD nomogram in the training cohort. Calibration curves depict the calibration of each model in terms of the agreement between the predicted risks of COVID-19 and observed outcomes of a confirmed diagnosis of COVID-19. The Y-axis represents the actual COVID-19 rate. The X-axis represents the predicted COVID-19 risk. The diagonal dotted line represents a perfect prediction by an ideal model. The black solid line represents the performance of the CIAAD nomogram, of which a closer fit to the diagonal dotted line represents a better prediction. **(C)**. Calibration curve of the CIAAD nomogram in the validation cohort. **(D)**. ROC curve of the CIAAD nomogram in the training cohort. **(E)**. ROC curve of the CIAAD nomogram in the validation cohort.

To simplify the model, the concrete values of CRP, PCT, and WBC were transformed into categorical variables (CRP and PCT: 1 for normal, 2 for high, 3 for undetermined; WBC: 1 for low, 2 for normal, and 3 for high), and fever was also defined as 0 for “no” and 1 for “yes.” Multivariate logistic regression analysis was performed with the five variables, and all of these potential predictors have substantial value with regard to distinguishing COVID-19 from infectious acute abdomen (Table 2). A risk score formula was preliminarily built to predict the probability of COVID-19 as follows: Logit (*P* = COVID-19) = 10.104 + 1.915 × fever + 1.753×chest CT + (−2.508) × CRP + (−0.8)×PCT + (−1.836) × WBC. The CIAAD nomogram was generated on the basis of the above result (Figure 3A).

**Table 2:** Multivariable logistic regression of potential predictors for screening COVID-19 in infectious acute abdomen patients (training cohort).

**Variables and Intercept**	β^*^	**Odds Ratio (95%CI)**	***P*-value**
Fever	1.915	6.788 (3.314–13.904)	<0.001
Chest CT	1.753	5.773 (3.172–10.507)	<0.001
CRP	−2.508	0.081 (0.043–0.152)	<0.001
PCT	−0.8	0.449 (0.281–0.739)	0.001
WBC	−1.836	0.160 (0.092–0.278)	<0.001
Intercept	10.104	

* *β: the regression coefficient*.

### Performance of the Nomogram in the Training and Validation Cohorts

The calibration curve of the CIAAD nomogram for the prediction of the risk of COVID-19 demonstrated good agreement between prediction and reality in the training cohort (Figure 3B). The C-index value for the prediction nomogram was 0.981 (95% CI, 0.963 to 0.999) in the training cohort. Good calibration was also observed in the validation cohort, with a C-index value of 0.966 (95% CI, 0.960 to 0.972) (Figure 3C). The ROC analysis in the training and validation cohorts yielded AUC values of 0.970 (95% CI, 0.961 to 0.982) and 0.966 (95% CI, 0.957 to 0.975), which suggested that the predictive performance was good (Figures 3D,E).

### Clinical Use and Development of a Simplified Scale

**Figure 4:**
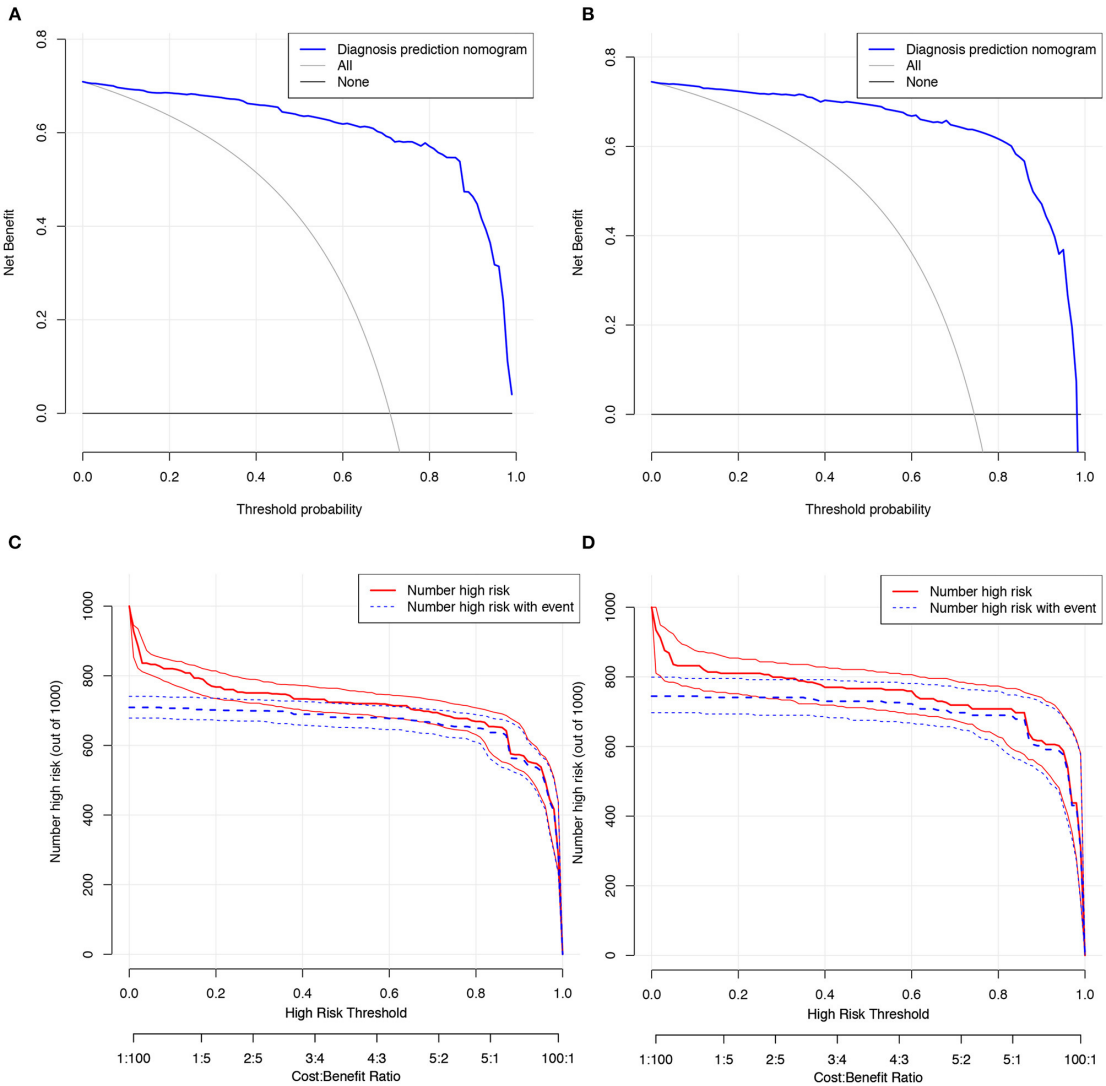
Decision curve analysis and clinical impact curves for the CIAAD nomogram in training and validation cohort. **(A)**. Decision curve analysis for CIAAD nomogram in the training cohort. The Y-axis measures the net benefit. The blue line represents the CIAAD nomogram. The gray line represents the assumption that all patients are COVID-19 patients. The black line represents the assumption that there are no COVID-19 patients. **(B)**. Decision curve analysis for the CIAAD nomogram in the validation cohort. **(C)**. Clinical impact curve for the CIAAD nomogram in the training cohort. The red curve (Number high risk) indicates the number of people classified as positive (high risk) by nomogram under each threshold probability. The blue curve (Number high risk with event) is the number of truly positive people under each threshold probability. **(D)**. Clinical impact curve for CIAAD nomogram in the validation cohort.

Decision curve analysis was performed and clinical impact curves were generated for the CIAAD nomogram in both the training and validation cohorts (Figure 4), demonstrating a high net clinical benefit that was almost greater than the entire threshold probability.

**Figure 5:**
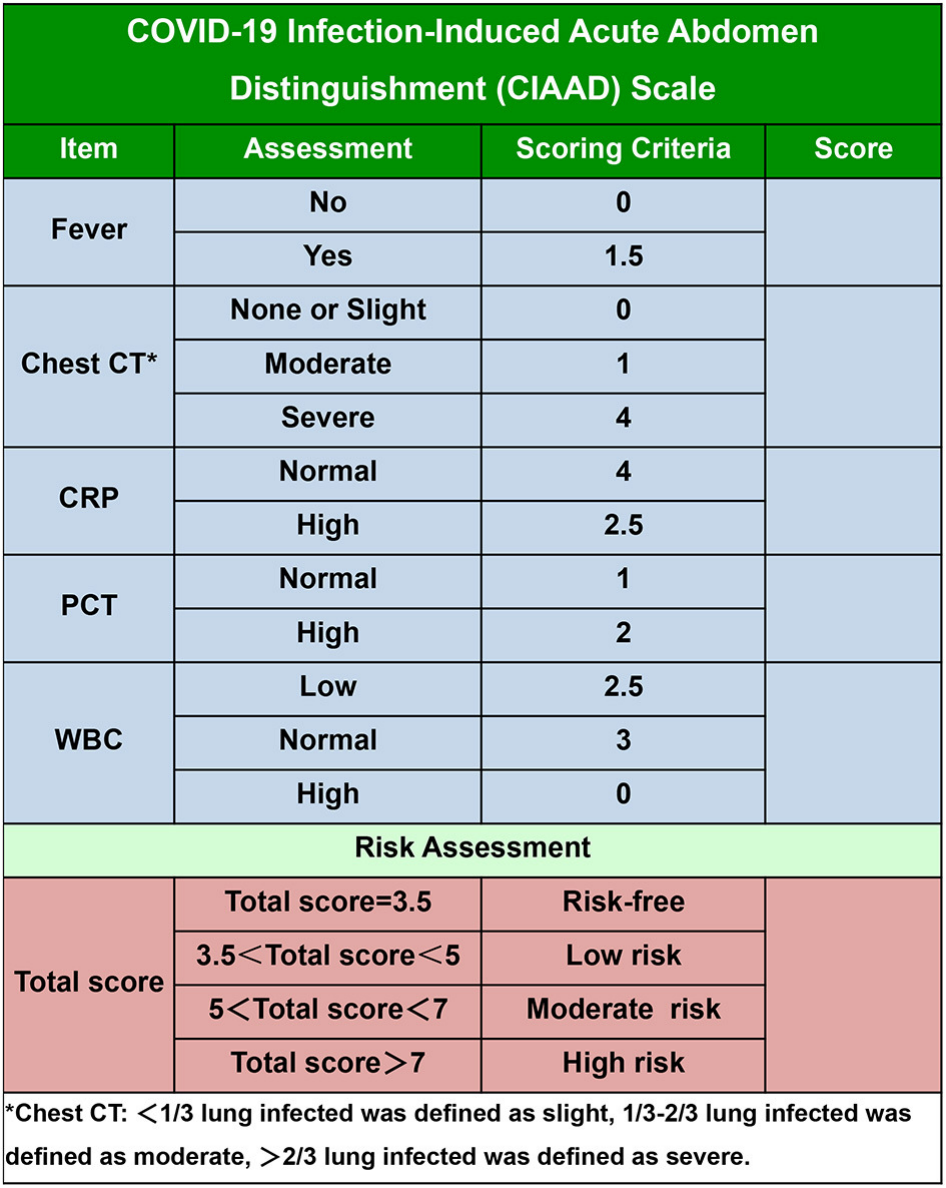
The COVID-19 Infectious Acute Abdomen Distinguishment Scale based on the CIAAD nomogram. Patients with a total score of <5 were considered of low risk of true SARS-CoV-2 infection, five to seven were of moderate risk, and more than seven meant high risk.

To make our prediction model more concise and practical in the context of emergency surgery, we simplified the scoring criterion of the CIAAD nomogram and created a new scale, named the CIAAD scale (Figure 5). The lowest and highest scores on this scale are 3.5 and 14.5, respectively. Items with higher scores are more common in COVID-19 patients, such as fever, abnormal chest CT, and normal levels of CRP and WBC. If the total score is 3.5, surgeons can regard the acute abdomen patient as COVID-19 risk-free. If the total score of a patient is <5, he/she has a low risk (<30%) of having confirmed COVID-19, and the risk increases to more than 70% as the total score reaches 7.

## Discussion

As the global outbreak of COVID-19 continues, the latest total of infected patients has exceeded 20 million, and humankind will face the threat of this disease for the foreseeable future (2). Vast amounts of medical resources have been expended to control the spread of the virus and treat affected individuals, resulting in the postponement of treatment for patients with many other diseases. However, for surgeons confronted with patients with infectious acute abdomen who urgently need to undergo emergency operations, it is necessary to accurately distinguish COVID-19 patients from those with similar and misguiding symptoms in the shortest possible time. To prevent the cross-infection of medical staff, such as doctors and nurses, and other patients in outpatient clinics, wards, and operating rooms, high-level precautions are necessary when managing patients who are strongly suspected of having COVID-19. Excessive precautions could waste substantial amounts of precious medical resources. In contrast, neglecting necessary screening would increase the risk of cross-infection of medical staff. The current screening procedures, such as nucleic acid detection and CT, have the disadvantages of inadequate accuracy and time-consuming operation. Consequently, a more convenient, efficient, economical, and effective COVID-19 screening method is desired by surgeons. To our knowledge, this study provides the first screening model and scale for surgeons to use to distinguish infectious acute abdomen patients from suspected COVID-19 patients in the emergency department by retrospectively comparing demographic, clinical, and laboratory characteristics of the two groups of patients. The CIAAD nomogram and scale have satisfactory performance with regard to the prediction of COVID-19 and great potential to help medical institutions resume routine medical work during the epidemic.

### Challenges and Opportunities

Current reports of COVID-19 patients show that respiratory symptoms, such as fever, cough, and dyspnoea, are the main clinical manifestations (10). Nevertheless, digestive symptoms, such as diarrhea, nausea, vomiting, and abdominal pain, are gradually being reported as early-onset symptoms, which deserves more attention (11, 12). A meta-analysis revealed that nearly half of the patients had positive results for SARS-CoV-2 in stool samples, and another bioinformatics analysis provided a probable theoretical basis for the digestive symptoms: angiotensin-converting enzyme II (ACE2) is highly expressed in the esophagus, ileum, and colon (13, 14). The mixture of fever and some digestive symptoms mimics the symptoms of infectious acute abdomen to a large extent. Similarly, if the patient is older or the abdominal infection develops into a systemic infection, signs of pneumonia can emerge in infectious acute abdomen patients as well. In patients with infectious acute abdomen, increased morbidity and mortality associated with a delay in the treatment of many of the surgical causes suggest the need for an aggressive and expeditious surgical approach (15). During the epidemic, quick and accurate screening of infectious acute abdomen patients suspected of having COVID-19 is vitally important.

The definite diagnosis of COVID-19 still mainly depends on a positive RT-PCR result for SARS-CoV-2 (16), which has the disadvantage of the possibility of false-negative results due to disease stage, viral load, and sample quality. In our COVID-19 cohort, the positive rate at the first nucleic acid test was merely 43.7% (255/584). Meanwhile, chest CT is also considered a good screening tool. However, the existence of mild cases not associated with pneumonia, atypical imaging findings, and substantial dependence on physicians' experience limit the screening value of chest CT. A study enrolled 1,014 COVID-19 patients from Wuhan and showed that the positive rate for chest CT was 88%, compared with 59% for RT-PCR8. Several prediction models based on the integration of demographic, clinical, imaging, and laboratory variables have been developed to evaluate the disease risk or prognosis (17). Unfortunately, the target populations of the published diagnostic models were patients presenting at fever clinics or ordinary patients suspected of having COVID-19 (17). It is not appropriate for surgeons to borrow these models directly to screen infectious acute abdomen patients, and our CIAAD nomogram and scale fill the existing gap.

### Strengths and Limitations of This Study

The strength of our model is the strong relevance. The recommended user of the CIAAD scale is a surgeon in the emergency department, and the recommended population to be assessed is infectious acute abdomen patients suspected of having COVID-19. To this end, we collected first-hand and high-quality data from COVID-19 patients and used strict enrolment criteria for infectious acute abdomen patients. In addition, via LASSO regression analysis, five quantifiable indicators were successfully selected. Although many variables, such as diabetes, cough, and D-dimer level, varied considerably between COVID-19 and infectious acute abdomen patients, they were ruled out by LASSO regression analysis as being too heavily weighted or causing the prediction model to be cumbersome. The selected indicators were all included in previous prediction models, which further verified the prediction capacity of these variables (17). We also clarified the differences of specific significant clinical indexes between COVID-19 patients and infectious acute abdomen patients. For example, COVID-19 patients often have no rise in CRP, PCT, and WBC, which was in accordance with the results of other studies. On the contrary, the infectious acute abdomen enrolled in our study suffered more severe inflammation, which resulted in more abnormality in the above-mentioned indexes. The discrepancy of infection indicators between two groups of patients truly reflected the difference in types of infection.

However, there was a limitation that cannot be evaded in our study. The disturbance of routine medical work by the epidemic resulted in the lack of patients with both COVID-19 and acute abdomen. As the number of emergency operations for acute abdomen decreased sharply in Wuhan, the data pertaining to acute abdomen patients were from B Hospital, a renowned hospital in China. We did not obtain patients with both COVID-19 and infectious acute abdomen, who would be the best study objects out of our research aim. The 822 patients were the most suitable two cohorts to gain distinguishment nomogram and scale. In spite of the limitation, patients with a score of 3.5 according to the CIAAD scale could be regarded as COVID-19 risk-free, which was of definite prediction accuracy.

### Implications for Practice and Future

**Figure 6:**
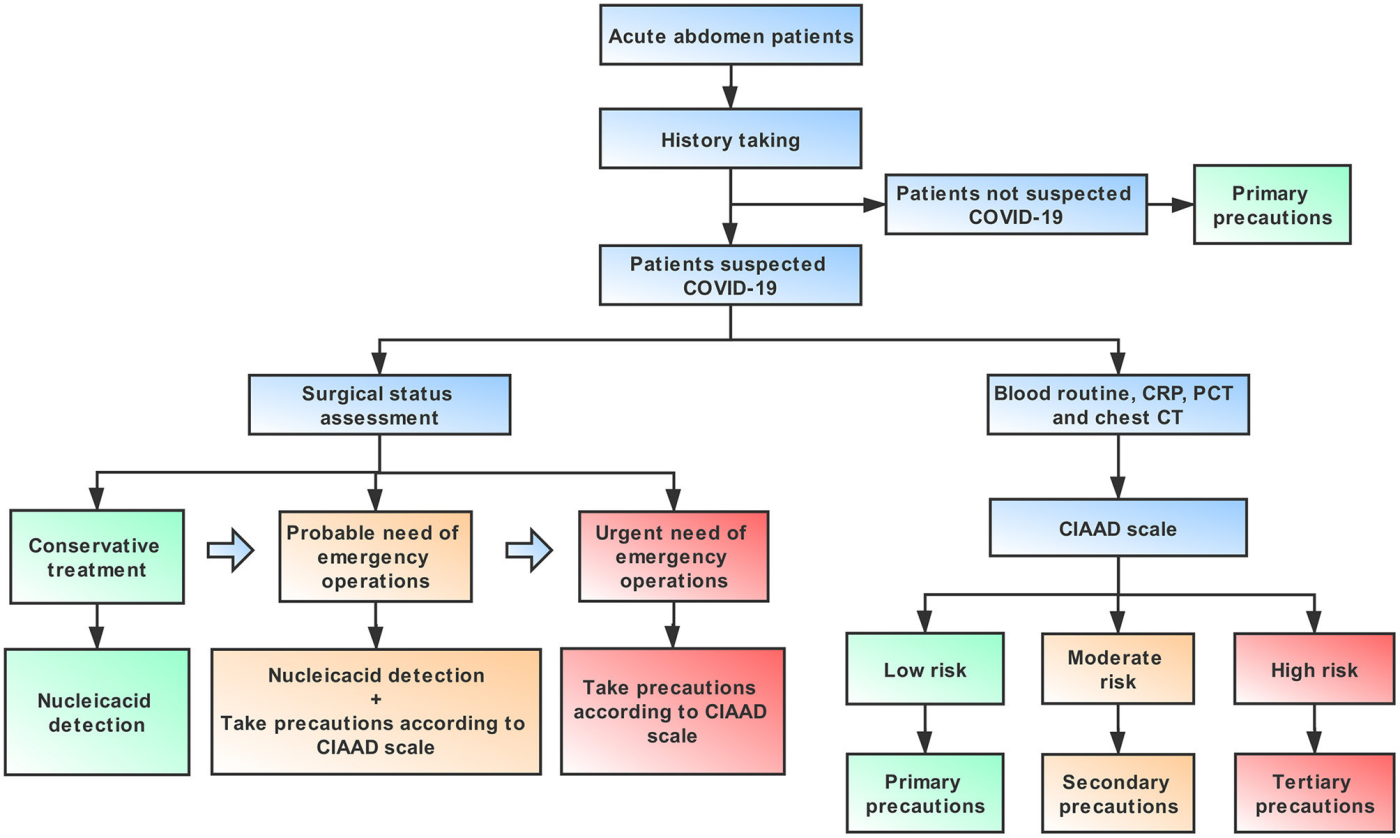
An algorithm help surgeons to manage infectious acute abdomen patients suspected COVID-19 in the emergency department (ED). Key procedures in this algorithm are history taking, surgical status assessment, and the CIAAD scale. Primary precautions are needed for all patients in ED during the epidemic.

An algorithm that is helpful for allowing both a focused workup and expeditious therapy is provided in this article, including necessary advice regarding prevention for medical staff, based on the guidelines published by the World Health Organization (18) (Figure 6). It is important to note that standard precautions are needed for all patients. For an infectious acute abdomen patient suspected of COVID-19, the first step is to evaluate his/her surgical status and assess the patient with the CIAAD scale. The degree of urgency of the patient's need for surgical intervention determines whether the medical staff should wait for the results of the nucleic acid test or take precautions according to our algorithm. The level of precautions adopted should be informed by the degree of risk of COVID-19 according to the results of screening with the CIAAD scale.

As the scale is harmless and has a net benefit over nearly the entire threshold probability, according to the decision analysis curves, we strongly recommend that surgeons worldwide use our CIAAD scale and the accompanying algorithm. With its wide use in a larger population, the efficacy of the CIAAD scale will be further prospectively tested.

## Conclusion

With the aim of distinguishing COVID-19 patients from infectious acute abdomen patients, we established an easy and effective screening model and scale for use by surgeons in the emergency department. The algorithm based on the CIAAD scale can help surgeons manage infectious acute abdomen patients suspected of having COVID-19 more efficiently and help prevent cross-infection.

## Data Availability Statement

The raw data supporting the conclusions of this article will be made available by the authors, without undue reservation.

## Ethics Statement

The studies involving human participants were reviewed and approved by The Ethics Committees of the Peking Union Medical College Hospital and the Central Hospital of Wuhan. The patients/participants provided their written informed consent to participate in this study.

## Author Contributions

ZBB, WYX, SWW, and QC contributed equally to this article. WWB, WYJ, ZBB, WYX, and SWW designed the study. ZBB, WYX, SWW, ZXT, LTH, CHT, and WWB collected assembled the data. ZBB, WYX, SWW, QC, and WZH analyzed and interpreted the data. ZBB, WYX, and SWW wrote the first draft, which all authors revised for critical content. All authors approved the final manuscript. WWB and WYJ are the guarantors. The corresponding authors attest that all listed authors meet authorship criteria and that no others meeting the criteria have been omitted.

## Conflict of Interest

The authors declare that the research was conducted in the absence of any commercial or financial relationships that could be construed as a potential conflict of interest.

## Abbreviations

ACE2: angiotensin-converting enzyme 2
AUC: area under the curve
CIAAD: COVID-19 infectious acute abdomen distinguishment
COVID-19: coronavirus disease 2019
CRP: C-reactive protein
CT: computed tomography
LASSO: least absolute shrinkage and selection operator
PCT: procalcitonin
ROC: receiver operating characteristic
RT-PCR: reverse transcription-polymerase chain reaction
SARS-CoV-2: severe acute respiratory syndrome coronavirus 2
WBC: white blood cell.
