# Supplementary figures and images for: Distinguishing Coronavirus Disease 2019 Patients From General Surgery Emergency Patients With the CIAAD Scale: Development and Validation of a Prediction Model Based on 822 Cases in China
Source: Front Med (Lausanne). 2021 Apr 30;8:601941. doi: 10.3389/fmed.2021.601941 (PMC8119634; doi:10.3389/fmed.2021.601941)

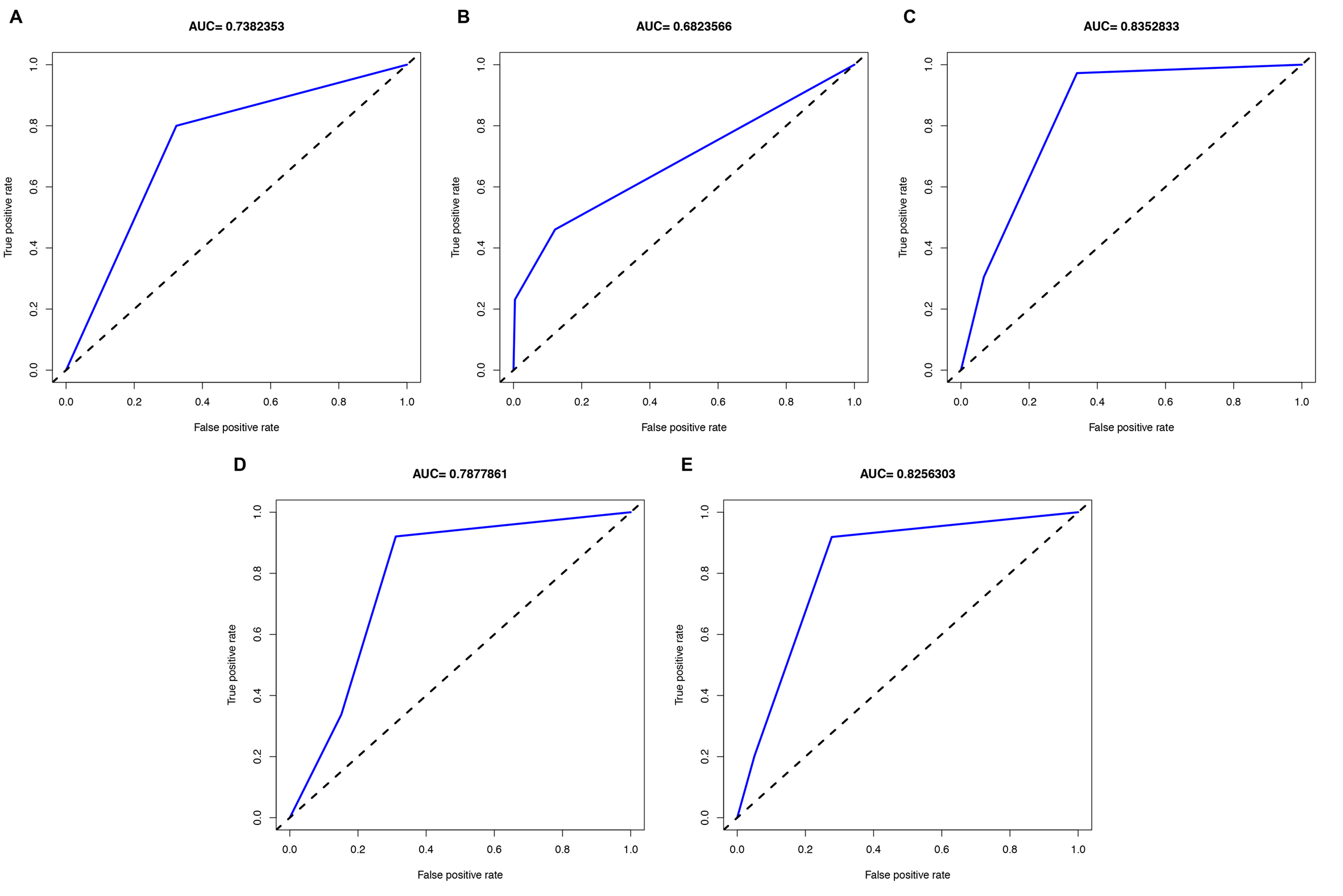

Supplement: Supplementary file 1 [file Image_1.TIF]

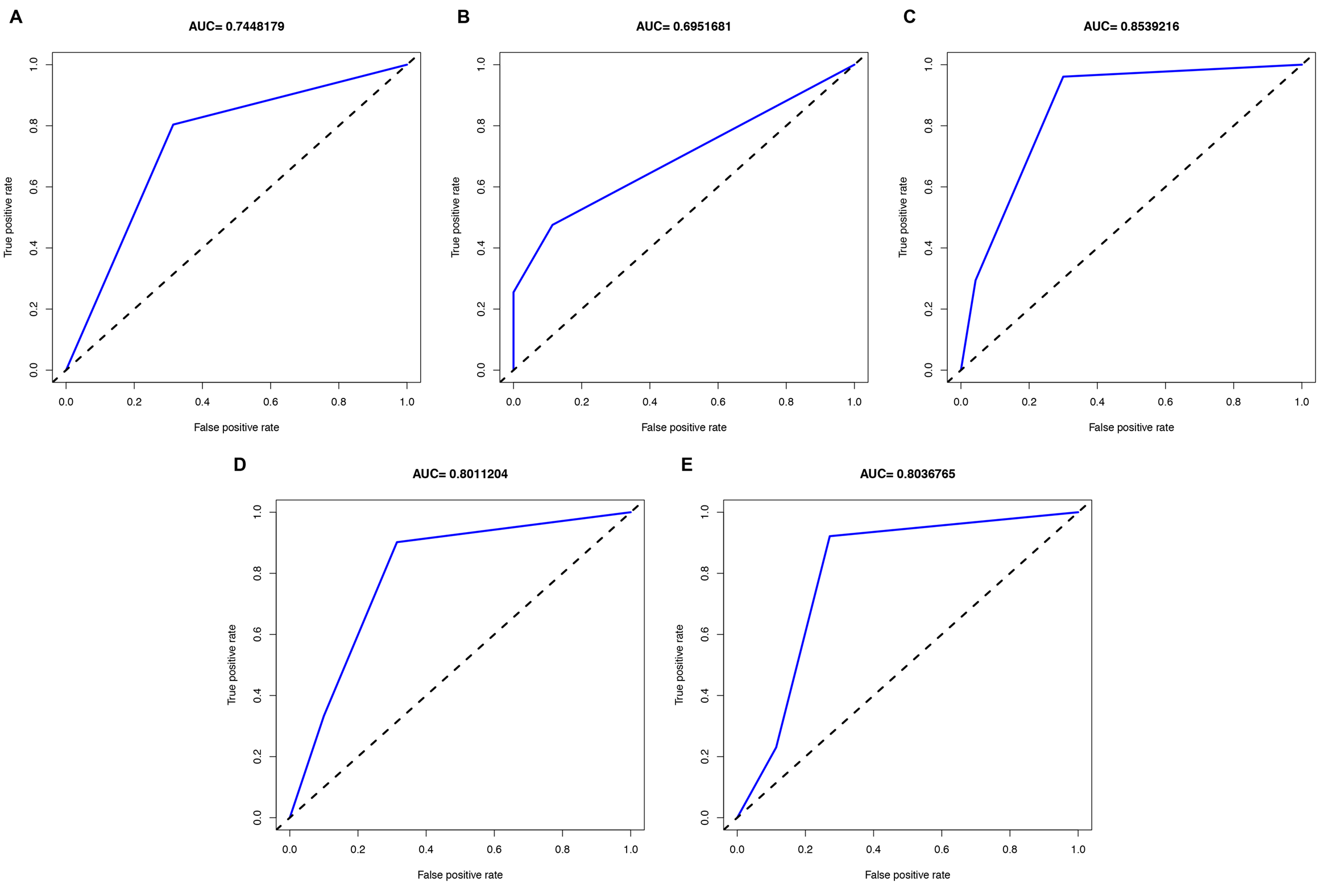

Supplement: Supplementary file 2 [file Image_2.TIF]
